# Dynamic Physiological Culture of Ex Vivo Human Tissue: A Systematic Review

**DOI:** 10.3390/cancers13122870

**Published:** 2021-06-08

**Authors:** Daniel Ll Hughes, Aron Hughes, Zahir Soonawalla, Somnath Mukherjee, Eric O’Neill

**Affiliations:** 1 Department of Oncology, University of Oxford, Oxford OX3 7DQ, UK; daniel.hughes@oncology.ox.ac.uk (D.L.H.); somnath.mukherjee@oncology.ox.ac.uk (S.M.); 2 Undergraduate Centre, Cardiff University Medical School, Cardiff CF14 4YS, UK; hughes.aron@yahoo.co.uk; 3 Department of Hepatobiliary and Pancreatic Surgery, Oxford University Hospitals NHS, Oxford OX3 7LE, UK; Zahir.Soonawalla@ouh.nhs.uk

**Keywords:** cancer, cell culture, dynamic physiological culture, perfusion culture, bioreactor, primary human tissue culture, organotypic tissue slice culture

## Abstract

**Simple Summary:**

Within cancer research, a strong emphasis is placed on the development of models that accurately reproduce the conditions in which tumours develop and grow. A limitation of several models is that they fail to replicate the tumour’s blood supply. Our aim was to evaluate the concurrent literature regarding dynamic physiological culture techniques that have been used to successfully culture human tissue. We conducted a systematic review of the literature and identified 22 articles that described the use of different dynamic culture techniques in order to create a system that was physiologically representative. The most common method described was the use of perfusion culture. This article serves as a detailed reference of novel technologies that can be implemented within cancer research in order to improve the physiological conditions of current culture techniques. Realistic cancer models will translate into a greater understanding of the disease which will directly impact on patient outcomes.

**Abstract:**

Conventional static culture fails to replicate the physiological conditions that exist in vivo. Recent advances in biomedical engineering have resulted in the creation of novel dynamic culturing systems that permit the recapitulation of normal physiological processes ex vivo. Whilst the physiological benefit for its use in the culture of two-dimensional cellular monolayer has been validated, its role in the context of primary human tissue culture has yet to be determined. This systematic review identified 22 articles that combined dynamic physiological culture techniques with primary human tissue culture. The most frequent method described (55%) utilised dynamic perfusion culture. A diverse range of primary human tissue was successfully cultured. The median duration of successful ex vivo culture of primary human tissue for all articles was eight days; however, a wide range was noted (5 h–60 days). Six articles (27%) reported successful culture of primary human tissue for greater than 20 days. This review illustrates the physiological benefit of combining dynamic culture with primary human tissue culture in both long-term culture success rates and preservation of native functionality of the tissue ex vivo. Further research efforts should focus on developing precise biochemical sensors that would allow for real-time monitoring and automated self-regulation of the culture system in order to maintain homeostasis. Combining these techniques allows the creation of an accurate system that can be used to gain a greater understanding of human physiology.

## 1. Introduction

When designing a culturing system, it is fundamental that it can reproduce the diverse in vivo conditions in order to replicate human physiology. Such a system would create an accurate platform that respects the dynamic physiological conditions required for homeostatic maintenance of both cells and organs, whilst preserving their functionality. Not only would it provide a unique opportunity to gain a greater understanding of in vivo biological processes of normal tissue, but it could also could be utilised in the context of studying human disease. For decades, the conventional technique and current standard of culturing cells are with static culture [1]. This adynamic method fails to respect the complex in vivo physiologic conditions due to the absence of perfusion (a proxy measure for blood flow), which would maintain an epistatic supply of nutrient delivery and allow for the removal of metabolic waste by-products [2]. Thus, static culture is hindered by its lack of ability to integrate several bio-physiological properties within its system.

As a consequence, much attention has been focused within the field of Bio-engineering on developing novel dynamic culturing systems that utilise and mimic normal physiological processes [3]. Several different dynamic culture systems have been described and published [4,5,6,7]. Whilst each system differs slightly, they all share a common theme that is that they all contain a dynamic element which recapitulates the properties of an in vivo physiological system. The nature of the in vivo physiological system replicated of course varies depending on the underlying biology of the desired experiment. In the context of a blood supply, perfusion delivery of media may be used, whereas dynamic pressure application serves as an option to assess mechano-transduction and load distribution when evaluating bone or articular cartilage [8,9]. This technology has been utilised to assess both normal and diseased conditions [5,10]. Emerging evidence from the published literature demonstrates the superiority of dynamic culture techniques over conventional static culture methods. Crabbe et al. demonstrated that the use of a rotating wall vessel bioreactor for decellularised murine lung tissue with lung cells and bone marrow-derived mesenchymal stromal cells (MSCs) resulted in significantly higher rates of proliferation and a decrease in apoptosis rates when compared to static culture [4]. The benefits of dynamic culture were also noted within the stromal compartment where higher rates of differentiation of MSCs into functioning fibroblasts were observed [4].

The use of the most sophisticated dynamic culture method or most advanced bioreactor design alone is insufficient to truly re-establish an in vivo physiological system, especially if using a single cell monolayer culture technique. The diverse cellular ecosystem is absent. In the context of cancer study, the cross talk between the cancer cell and its microenvironment is an essential component of the cell survival mechanism [11]. Therefore, the use of homogenous and immortalised cell lines derived from a human tumour lacks the microenvironment context and subsequent symbiotic relationship. For these reasons, primary human tissue culture serves as an opportunity to preserve the multi-cellular component and the surrounding architecture [12]. In light of the nuance of dynamic culture technology, its role in primary tissue culture has yet to be determined. One could hypothesise that combining dynamic culture techniques with primary human tissue culture would create an advanced platform on which physiology and multi cellular scaffolds are preserved. The aim of this systematic review is to critically analyse the current literature regarding the use of dynamic culture techniques with ex vivo human tissue in order to determine whether long-term culture is possible when physiological processes are preserved.

## 2. Methods

This systematic review was conducted with adherence to Preferred Reporting Items for Systematic Reviews and Meta-Analyses (PRISMA) guidance [13]. A comprehensive and detailed search through the current literature was performed. Three online databases were searched individually (PubMed, Medline and EMBASE) for appropriate articles published between 1946 and 2020. A specific search strategy was developed that combined human tissue culture and dynamic physiological culture. Specific search terminology included tissue explants, explants, tissue slice, human tissue, organotypic tissue slice for primary human tissue culture and microfluidics techniques, perfusion culture, perfusion bioreactor, bioreactor, and microfluidics for dynamic physiological culture, respectively. The strategy was implemented through a title search combined with MeSH search terms. Both of the Boolean operators (AND or OR) were incorporated into the search in order to facilitate maximum article capture. The final search was supplemented by manual hand searching of reference lists of both included and excluded papers. All search results were combined using Rayyan software where duplicate references were removed [14].

Two authors (D.H. and A.H.) conducted independent screening of all the titles and abstracts from the final search against specific inclusion and exclusion criteria. Our inclusion criteria consisted of two conditions. Firstly, all included articles must use primary human tissue for ex vivo culture for their experiments. Tissue acquisition may be obtained through surgical resection, biopsy or from post mortem analysis. Secondly, included articles must use dynamic physiological culture methods. We defined dynamic physiological culture as any culturing method that incorporated a dynamic, kinetic energy component to the culture system in order to replicate an element of normal in vivo physiology. Any physiological system could be replicated, for example blood flow via perfusion or pressure application for mechano-transduction. Conventional culturing techniques i.e., static culture are adynamic and do not replicate normal physiology; therefore, any article solely using static culture methods was excluded. Our exclusion criteria consisted of any article using monolayer two-dimensional cell culture, animal tissue, decellularised tissue or a lack of a dynamic physiological culture system. Articles that described immortalising the tissue (such as fixing in formalin) prior to dynamic physiological culture were excluded. Non-full text and non-English articles were excluded. 

Data extraction was performed by two authors (D.H. and A.H.) of the included articles using a predesigned data collection proforma. Any disparities were resolved by a consensus. Data collection regarding dynamic physiological culture methods included a description of the novel system, the nature and the numeric values of representative physiology being replicated. Data obtained regarding primary human tissue culture included: method of tissue acquisition, thickness of tissue, length of successful ex vivo culture and whether functionality of the native tissue was preserved. Our primary outcome measure was to determine what novel dynamic physiological culture methods had been previously described and what physiological component had been replicated ex vivo. Our secondary outcome measure was to determine whether the use of dynamic physiological culture for human tissue resulted in a greater duration of ex vivo culture where the tissue retained its native function. In light of the heterogeneity of the data, no formal meta-analysis was undertaken. No ethical approval was required for this systematic review.

## 3. Results

### 3.1. Literature Search 

**Figure 1:**
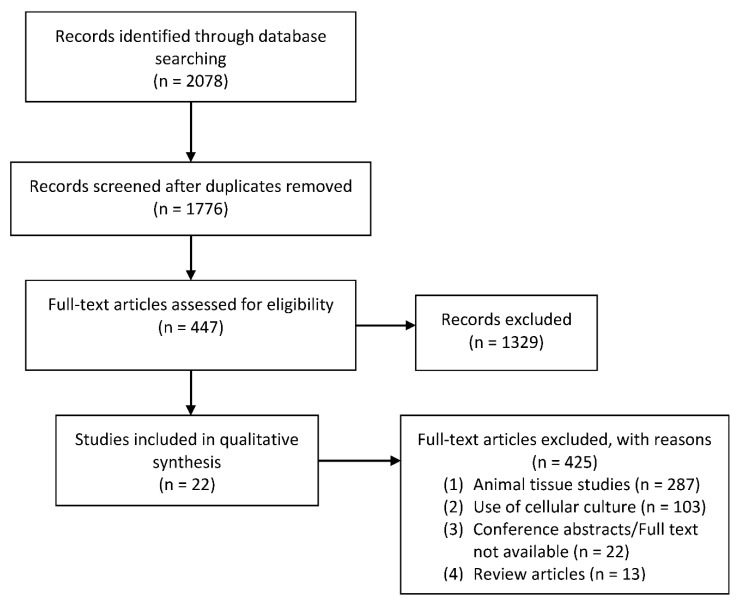
PRISMA flow diagram of search strategy and included articles.

Following a thorough and systematic search through the concurrent published literature, a total of 2078 articles were identified. After a vigorous screening process with cross referencing to the specific study inclusion and exclusion criteria, a total of 22 studies were identified and included for qualitative synthesis (Figure 1) [15,16,17,18,19,20,21,22,23,24,25,26,27,28,29,30,31,32,33,34,35,36]. The use of animal tissue for ex vivo culture was the most frequent reason for exclusion (287 articles), followed by primary cell culture combined with dynamic physiological culture (103 articles). 

The initial analysis consisted of a critical appraisal of the described technology used to establish dynamic physiological culture within each included article. Whilst several different techniques were described, it became apparent that three recurring themes emerged that could group the dynamic physiological culture systems according to the method used to replicate in vivo physiological conditions. These themes were the use of dynamic mechano-transduction, rotational bioreactor and dynamic perfusion. 

### 3.2. Mechano-Transduction Culture Models

**Figure 2:**
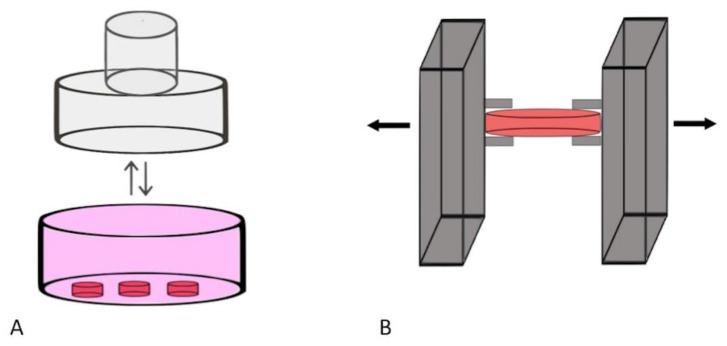
Dynamic physiological culture techniques that replicate dynamic mechano-transduction physiology. (**A**) an illustrative example of the dynamic cyclic compression technique. A vertical pressure load is applied to the cultured tissue. The loading weight applied can be altered in addition to whether the pressure load remains constant over a sustained period of time or is intermittent in nature. The pink component of the image is representative of the media within the culture plate, whereas the small red cylinders portray the cultured tissue; (**B**) an illustrative example of the mechanical expansion and stretching technique. The tissue is initially secured and subsequently a uniaxial stretch load is directly applied to the tissue. Applied tension can either be sustained and progressive or episodic. The red cylinder represents the cultured tissue.

Four articles utilised a pressure mechano-transduction model to establish dynamic physiological culture (Figure 2) (Table 1) [15,16,17,18]. These systems incorporated a motor element that delivered pressure forces on to the cultured tissue. The pressure forces applied were created either through the direct pressure application on the tissue or through fixing the tissue with subsequent implementation of longitudinal stretch [15,16,17,18]. Naturally, human tissue cultured under these conditions was selected based on the fact that their native biological function was to sense mechanotransduction or to sustain shear forces. Of these articles, three assessed the impact of pressure load on bone or intervertebral disc culture [15,17,18]. The remaining study evaluated the effect on skin (via the culture of a juvenile prepuce) [16]. The direction of the pressure load applied varied according to the tissue being cultured. All three studies that cultured bone/intervertebral disc applied a vertical pressure load, whereas a horizontal uniaxial stretching load was used when culturing skin [15,16,17,18]. The amount of the pressure load delivered varied between studies as did the applied duration of the force on the tissue. All four articles constructed their dynamic physiological culture technique within their own lab [15,16,17,18]. The experimental design consisted of investigating as to whether the ex vivo tissue was able to withstand and respond to the delivered pressure load. Cultured bone was frequently measured (both in terms of height and weight) following the application of pressure. Culture viability was assessed and presented as proportion alive (% total cells) following DAPi and MTT or Calcein AM staining [15,18].

### 3.3. Dynamic Culture with a Rotational Bioreactor

**Figure 3:**
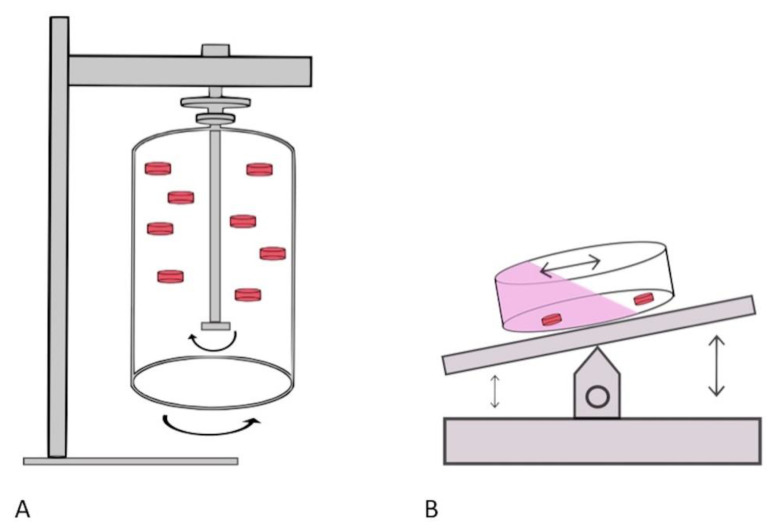
Dynamic physiological culture techniques that utilises a rotational bioreactor to replicate physiological flow dynamics. (**A**) An illustration of one method whereby tissue is suspended in culture medium due to concurrent rotation of both inner rotating cylinder and outer vessel wall. As a consequence, the tissues (illustrated by the red cylinders) are maintained in a state of continuous free fall with minimal shear stress exerted on them; (**B**) an illustration of how a rocking platform bioreactor enables dynamic culture. The serial rocking movement permits the flow of media (illustrated in pink within the figure) over the cultured tissue. The preexisting culture media is redistributed across the culturing vessel. However, there is no automated renewal of the media.

A rotational bioreactor was used as a dynamic physiological culture technique in six articles [19,20,21,22,23,24]. The method was defined by its use of a rocking/rotational movement (Figure 3). A diverse range of tissue (both normal and diseased) was cultured ex vivo using this technique (prostate, melanoma, tonsil, bone marrow, colon and liver tissue) [19,20,21,22,23,24]. A detailed description of each system is recorded in Table 2. The most frequent technique used (described in 4/6 articles) incorporated the use of rotating wall bioreactors that allowed dynamic movement which enabled the cultured tissue to be in a continuous state of ‘free fall’ [19,20,21,22]. Being suspended in such a state resulted in low shear stress conditions and favoured mass transfer of nutrients. Three articles specified a rotation speed (ranging from 20–40 rpm), whereas the remaining articles simply used continuous rocking [19,20,21]. The rotational bioreactor method utilised a large volume of culturing media in their culturing vessel. Whilst two articles reported culturing media volume of less than 3.5 mls, the majority (67%) used significantly greater culturing volume ranging from 10–200 mls (mean 80 mls). In light of the large volume used within the culturing vessel, the time interval between media replenishment was determined. Two different approaches were noted. Durray et al. (2005) and Paish et al. (2019) reported frequent changing of the media (at a three day and one day interval, respectively) [21,24]. However, both Margolis et al. and Licat et al. performed a media change every seven days, their total culturing volume was 55 mls [19,20]. Of the articles that described the use of a bioreactor for ex vivo tissue culture, none had the capability of auto replenishment of the culture media. All were dependent on manual media renewal.

### 3.4. Dynamic Perfusion Culture

**Figure 4:**
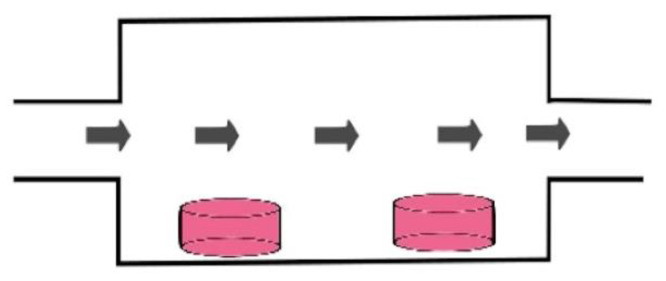
Dynamic perfusion culture. An illustration of dynamic perfusion culture technique. Active inflow of media into the bioreactor is driven by a mechanical pump. The inflow can be continuous or intermittent. An outflow exit channel is required in order to prevent volume overload of the system. Outflow management may be passive in nature or may require active draining through a pumping mechanism. The red cylinders represent the cultured tissue. Adjacent culturing wells can be implemented in order to facilitate multi tissue culture. Further modifications to the system include a scaffold for tissue support and a channel for drug delivery.

The final dynamic physiological culture technique noted in this systematic review was the use of dynamic perfusion (Figure 4) (Table 3) [25,26,27,28,29,30,31,32,33,34,35]. This was the most frequently implemented method of dynamic culture within the included articles (55%). Dynamic perfusion was performed on both a micro and macro scale. The flow rate exceeded 100 mL/min for three articles [25,27,33]. A correlation was noted between the tissue cultured and the delivered flow rates. Culture of tissue derived from the cardiovascular system (heart, vein, etc.) resulted in the use of much higher perfusion flow rates. In the context of perfusion culture, the nature of the perfusion regime (continuous or intermittent) serves as an important variable that influences nutrient delivery. Within this systematic review, perfusion with an intermittent flow was the most frequent technique adopted (32%) [25,26,27,29,30,33,34]. Establishing perfusion flow requires a driving force, therefore a pumping mechanism is utilised in order to generate kinetic energy. A peristaltic pump was the most common method used to deliver flow (23%), followed by a syringe pump (18%). An important component of a perfusion culturing system is the management of outflow waste. Two distinct approaches can be utilised; passive outflow management relies on unsupported drainage of the system where drainage is achieved by wide bore outflow channels and gravitational pull. Such an approach may not be suitable for systems with high flow rates and small volume chambers. Failing to match the outflow and inflow rates will result in excessive delivery of media to the system with subsequent flooding. Active outflow management is facilitated by the use of a pumping device attached on to the drainage channel which actively removes media from the system, thus higher drainage rates can be achieved. Passive outflow management was described in 32% of the included dynamic perfusion articles [26,28,31,32,33,34].

### 3.5. Successful Culture of Ex Vivo Human Tissue with Dynamic Culture Techniques

This systematic review subsequently analysed which human tissue had been successfully cultured ex vivo with dynamic physiological culture techniques. Tissues were grouped according to broad organ systems. This included cardiovascular, musculoskeletal, genito-urinary, gastrointestinal system and miscellaneous (Table 4). The genito-urinary system tissues were most frequently cultured with dynamic physiological culture (6/22, 27%), followed by gastrointestinal tissue (5/22, 23%). The median duration of successful ex vivo culture of primary human tissue for all articles was 8 days; however, a wide range was noted (5 h–60 days). Six articles (27%) reported successful culture of primary human tissue for greater than 20 days. These included cultured articular cartilage, intervertebral disc, bone marrow, prostate tissue, testicular tissue and breast cancer tissue. The longest duration of successful ex vivo culture was reported by Perrard et al., in which testicular tissue was successfully cultured for 60 days [31]. Tissue functionality was preserved ex vivo with continuous spermatogenesis detected by fluorescence in situ hybridisation identification of haploid cells at day 60. Of all tissue cultured ex vivo, tissue from the musculoskeletal system was cultured for the greatest duration (median 21-day range 10–56 days).

Both normal and diseased tissue were successfully cultured ex vivo with dynamic physiological culture techniques. A significantly higher proportion of the included articles cultured normal tissue ex vivo (15/22, 68%), thus reflective of the perceived importance of establishing physiological conditions when culturing primary human tissue ex vivo. It has previously been reported that the time interval between tissue acquisition and culture should be minimal in order to preserve tissue integrity and reduce cellular ischaemic injury. We therefore investigated the reported time metrics of the tissue transition/cellular ischaemic injury window within the included articles. Tissue processing was rapid and in 45% of the articles, occurred within 2 h of tissue acquisition. Several different techniques were described with regard to tissue sample preparation/tissue slice creation. Manual dissection of the tissue served as the most frequent method for tissue preparation (8/22, 36%), followed by vibratome tissue slice creation (5/22, 23%). Culture conditions, specifically media composition and supplementation, varied significantly between tissue types (Supplementary Table S1). Antibiotic prophylaxis against infection was utilised by 50% of the included articles. Of the 11 articles that utilised routine antibiotic supplementation in the media, 100% reported a multi-drug regime for antimicrobial cover. Tissue thickness serves as an important parameter that can impact long-term culture rates. Oxygen permeation through tissue is hindered if the tissue is of significant thickness. As observed in this systematic review, tissue thickness was kept to a minimum across all studies. 

Determining the viability of cultured primary human tissue serves as an important component for any ex vivo culturing system. What is clear from this systematic review is that several different techniques can be implemented as a read out for viability. In 64% of the included articles, histological assessment was utilised to assess tissue morphology. Further staining (either with immunochemistry or fluorescent staining) provides a unique opportunity to quantify active biological processes within the tissue, notably cell proliferation or apoptosis rates. In addition to determining viability, assessing whether native tissue function is maintained ex vivo is an essential factor that determines the efficacy of primary human tissue culture. Of the 22 included articles, 12 (55%) formally assessed native tissue function, examples of which include bile acid synthesis, albumin secretion, hormone production, response to mechanical load and complex biological processes such as spermatogenesis. 

## 4. Discussion

Recent technological advances have resulted in the development of next generation culturing systems. These systems incorporate a dynamic, kinetic element to re-establish an in vivo physiological process. What is clear from this systematic review is that, despite the nuance of this technology and its apparent physiological benefit, it has not yet been widely adopted as a culturing technique within the scientific research community. This is apparent within this review as only 22 articles were noted to combine primary human tissue culture with dynamic physiological culture. The technology utilised is dependent on the nature of the physiological process being replicated. To mimic the mechanobiology of a pressure load distribution across an articular surface, an axial loading compression cylinder should be incorporated into the culturing system, whereas utilising microfluidic perfusion may recapitulate tissue blood supply. When designing such a system, physiology should influence and determine the construct in order to create a dynamic physiological culture technique. 

Since the first published description of static monolayer cell culture, this technique has served as the gold standard culturing method for over a century [37,38]. Whilst this technique clearly serves a purpose, one should be cautious of the fact that it is not physiologically representative of in vivo conditions. In vivo tissues have an ample vascular capillary network that provide nutrients and oxygen required for cellular biological processes. Ex vivo, tissue and tissue constructs are devoid of a capillary network and are therefore dependent on passive diffusion of oxygen and nutrients into cells [39]. The diffusion limit of oxygen is approximately 200 μm, thus cells that are outside this limit are nutritionally starved within static culture [39]. Nutritional deprivation has been demonstrated to have an adverse impact on cellular proliferative capacity and new matrix deposition [40]. Malda et al. further demonstrated that nutrient deprivation resulted in non-uniform matrix deposition with reduced quantity centrally compared to peripherally in tissue constructs [41,42]. Thus, it illustrates the impact of regional nutritional depletion on biological processes. Within this systematic review, we observed that tissue thickness was kept to a minimum in order to facilitate maximum oxygen diffusion. The optimal tissue thickness will vary according to the type of tissue being cultured and the design of the culturing system.

The ability to perfuse media on a microscale (microfluidics) has significantly advanced over the last decade. Combining microfluidic perfusion with cellular culture has led to the development of an “Organ On a Chip” (OOC) platform [43,44,45,46]. This approach allows the establishment of a functional physiological unit ex vivo that can be assessed and manipulated [43]. Since the first description of OOC in 2010 (a heart lung model), the technology has rapidly evolved and the complexity of the chip platform has been augmented either by introducing multiple tissue components or different physiological stimuli [46]. As highlighted in the review article by Low et al., OOC technology may be of particular benefit in the context of drug discovery notably when assessing off target side effects and drug toxicity profiles [46]. The versatility of the OOC platform enables users not only to study the mechanism of disease (for example, metastatic dissemination or immune cell infiltration) but also permits the study of normal human physiological process (such as the gut hormonal axis or stem cell differentiation) [46]. As illustrated in the review of Wu et al., OOC systems may vary between manufacturers and models; however, they all possess the same fundamental design concepts [47]. These include the ability to culture organic material (be that cells or tissue), the presence of a dynamic element that introduces kinetic energy to the system (most frequently this is microfluidic flow) and, finally, the capability of drug delivery and response detection [47]. The system may require further modification specifically in relation to the nature of the tissue or cells being cultured, the flow rate, the air–fluid interface and to whether a support scaffold is required [47]. To date, an OOC for liver, lung, intestine, heart and kidney have been described [47]. The complexity of the platform can be further augmented by the co-culture of multiple cell lines or tissue samples. Tsamandouras et al. published their experience of co-culturing gut and liver on their OOC [45]. They investigated the pharmacokinetic changes following drug delivery in the context of multi organ culture [45]. The ultimate aim of OOC technology is to upscale the capacity of the system so that multiple tissues can be co-cultured, with the penultimate objective of being able to produce a “human on chip” [47]. To date, the most comprehensive system included 10 organs within their platform [48]. In addition to assessing organ function (either through measuring synthetic function such as C-peptide production by the pancreas or through transepithelial/transendothelial electrical resistance measurements on skin), the authors were also able to review diclofenac metabolism within all organs [48]. Several other groups have since published on the benefit of multi organ culture; however, these have predominantly been in the setting of monocellular culture [45,48,49,50]. The adoption of OOC technologies with primary human tissue has yet to be widely implemented. This systematic review only identified 11 articles that utilised dynamic perfusion (via a pump delivery system) and primary human tissue. A possible explanation for this is that of these 11 articles, 10 of which (91%) had constructed their OOC platform within their own laboratory ‘in-house’. The lack of access to commercially available OOC platforms may therefore prevent its widespread use. 

Emerging literature has illustrated the benefit of culturing organoids derived from human tissue within a microfluidic system [51,52]. Schuster et al. derived organoids from patients with pancreatic cancer and conducted a high throughput drug screen [52]. Published protocols for organoid creation require an initial tissue dissociation step followed by enzymatic digestion prior to organoid establishment and culture [53,54]. It remains difficult to determine to what extent the entirety of the primary tissue has been successfully recapitulated ex vivo within the organoid with this multistep creation approach. The tumour microenvironment is a diverse multicellular ecosystem, and it is unclear to what extent this is reproduced within the organoid. Within this review, we defined a primary tissue culture as any technique that performed minimal modification or processing steps to the tissue after its acquisition and prior to culture. It was felt that this would serve as a more reliable definition of primary tissue and as such would reduce the heterogeneity of the included articles. Therefore, organoid culture was not included within the systematic review. Several comprehensive literature reviews regarding microfluidic organoid culture have been previously published [55,56].

One important aspect to consider regarding the biochemical cell response to mechanical stimuli is the role of the ubiquitous second messenger, calcium. The intracellular calcium ion (Ca^2+^) concentration has been demonstrated to be highly involved in several physiological signaling pathways and to be sensitive to mechanical signals [57]. None of the articles in this review investigated the role of Ca^2+^ among human tissue cultured with dynamic physiological culture techniques. Further research surrounding the role of Ca^2+^ within human tissue cultured among these dynamic conditions is therefore needed to allow for a better appreciation of their representation of real physiological conditions in terms of mechanical stimulation of tissues and cells.

For any culturing system, an important consideration is nutrient delivery to the tissue. Within static culture, a finite concentration of nutrients is present. Over time, as a consequence of proliferation and active cellular biological processes, the availability of nutrients is diminished. Media replacement causes a harsh environmental change and a sudden transformation from a nutrient deplete to nutrient excess setting which can precipitate cellular stress [58]. Garcia-Montero et al. demonstrated that media exchange results in upregulation of stress activated genes such as p38, ERK1/2 and JNK [58]. A nutritional concentration gradient may be apparent within static culture, whereby a higher availability is present on the periphery of the tissue when compared to the centre [58]. As a consequence, central necrosis may develop and geo-spatial variation in tissue function may occur [59]. With such limitations in static culture, it is clear that perfusion culture may overcome such issues. Within this systematic review, it was noted that the vast majority of dynamic physiological culture techniques (77%) incorporated continuous media exchange within their system, either through a constant epistatic supply via a perfusion pump (11/22) or through rotational movement to ensure mixing of nutrients (6/22). This highlights the perceived importance of nutrient delivery in primary human tissue culture.

Tissue acquisition may serve as a rate limiting step. The window of cellular ischaemic injury (time interval between procurement and culture) should be kept to a minimum. Within this review, a tissue culture occurred within 2 h of tissue acquisition in 45% of the included articles. Tissue preparation techniques varied across studies. Manual dissection was the most frequent method utilized; however, the reproducibility and the creation of homogenous consecutive tissue slices with this technique should be queried. Culturing media was heavily supplemented with numerous growth factors, antibiotics and amino acid derivatives (Supplementary Table S1). The creation of universal tissue specific culturing medium may allow consistency between studies and comparability of successful long-term culture rates. 

There are limitations to note within this systematic review. The included articles are highly heterogenous, therefore a formal meta-analysis was not conducted. Whilst several different primary human tissues were cultured, only a minority of tissue was duplicated; thus, a formal comparison of duration of successful ex vivo culture amongst the same tissue type was not possible. Significant variation was noted in the constitution of the culturing media utilised between studies, which may directly impact on successful culture duration. The majority of the included articles (64%) had constructed their dynamic physiological culture system internally (“in house”), thus assessing the reproducibility of the culturing methods described, and their results remain to be determined within a broader context.

As the field of biomedical engineering continues to advance at a marked speed, research efforts should be focused on developing and validating novel culturing methods that are commercially available at a reasonable cost. The high price of material acquisition for system creation or commercial purchase limits the availability of this technology for wider use within the research community. Further efforts should ensure that a reasonable price per unit is feasible with reusable components. As there is a marked number of culture models created internally within laboratory groups, there is a need to develop standardised reporting criteria, both in terms of system design and function in addition to functional viability assessments. Implementing an international registry would ensure homogeneity of the literature, facilitate transparency of data and assure reproducibility of culture experiments. The ability to culture multiple organs concurrently may require specialised universal media. Perhaps of most importance is the development of robust sensory monitors within these culture systems [60]. Incorporating accurate sensors within this technology would facilitate real-time monitoring of the culturing environment. This would provide an opportunity to intervene early and change or alter the culturing conditions as required. It would facilitate the correction of electrolyte or nutrient imbalance within the culturing system. Such automated monitoring would ensure regulation and maintenance of homeostatic culture conditions. Combining high throughput sensors with communication software also raises the possibility of being able to have remote monitoring capability. The use of artificial based intelligence software would allow the culture system to self regulate and would be a significant step closer to creating a virtual patient.

## 5. Conclusions

Combining dynamic physiological culture techniques with primary human tissue culture serves as a unique opportunity to establish a physiologically representative culture system. This systematic review demonstrated that the wider implications of this technology are yet to be implemented in research practice. The nature of the physiological process being replicated ex vivo will dictate the design of the culturing system. Successful long-term culture ex vivo of a range of primary human tissue was noted. Recognising and replicating the normal physiological processes that govern tissue viability allowed the preservation of native tissue function. Further research is required to determine the optimal culture conditions and physiological parameters for tissue specific experiments. Utilising both dynamic physiological culture techniques and primary human tissue culture provides an exclusive opportunity to further study human physiology in the ex vivo setting.

## Figures and Tables

**Table 1:** Characteristics of studies utilising dynamic physiological culture to replicate dynamic mechano-transduction physiology.

Author	Tissue Cultured	Nature of Physiology Being Replicated	Direction of Load	Loading Weight Applied	Characteristics of Applied Loading Force	Duration of Force Applied	Recovery Time	Number of Samples Cultured per Bioreactor
Rosenzweig, D. (2000) [15]	Intervertebral Disc	Dynamic cyclic compression	Vertical	Loads were cycled 0.1 MPa and 0.3, 0.6 or 1.2 MPa	Dynamic loading	2 periods of 2 h per each load	Periods of 6 and 14 h, maintaining a low-static load (0.1 MPa)	One sample fits entirely into one independent bioreactor (1 of 3 units)
Ladd. M. (2016) [16]	Juvenile prepuce	Mechanical expansion/stretch	Uniaxial stretch	Expansion of the skin by 20% of its initial length per day for 5 days to double the original length	Dynamic stretching	5 days. Progressive stretch maintained until target maintained	Maintenance of each stretch for 55 min	One sample per bioreactor unit
Aiyangar, A. (2017) [17]	Trabecular bone (L1 vertebrae)	Dynamic cyclic compression	Vertical	10 N or 20 N	Dynamic loading	NS	Unloading period after each compression	One sample per bioreactor unit
Walter, B. (2017) [18]	Intervertebral Disc	Dynamic cyclic compression	Vertical	0.1 MPa/0.2 MPa	Dynamic and static loading	12 h for each load for 7, 14 or 21 days	No recovery time	Able to load 6 or 9 samples simultaneously

NS—Not specified, N—Newton, MPa—MegaPascal.

**Table 2:** Characteristics of studies utilising a rotational bioreactor with physiological flow dynamics.

Author	Tissue Cultured	Description of Bioreactor	Rotation Speed	Time Interval for Media Exchange	Volume of Media within a Bioreactor
Margolis, L. (1999) [19]	Prostatic tissue	Cultured tissue is suspended in culturing liquid medium enclosed by an inner and outer rotating cylinder. The tissue and medium rotate in unison under low shear force. The rotation results in an equilibrium between gravitational-induced sedimentation of the tissue/cells and a centrifugal force.	30 Rpm	Bioreactor medium was 50% renewed every 7 days	55 mL
Licato, L. (2001) [20]	Melanoma tissue	Rotating-wall vessel bioreactor is completely fluid filled. Specific rotation around a horizontal axis occurs that results in the cultured tissue or cells to be in a state of continuous free fall under low shear stress conditions, designed for mass transfer of nutrients.	20 Rpm	Media replaced once a week. Bubbles were removed from the vessels daily, so that the chamber remained completely fluid filled	55 mL
Durray, P. (2005) [21]	Tonsillar tissue	Same method and technique as described by Margolis (1999)	35–40 Rpm	Media sampled every 3 days and replaced.	200 mL
Ferrarini, P. (2013) [22]	Bone marrow	Horizontally rotating bioreactor utilised in order to create a laminar flow (Rotatory Cell Culture System). Cultured 3D tissues suspended in a “free falling” position in order to minimise turbulence and shear forces across the tissue. Gas exchange membrane incorporated in vessels to ensure optimal oxygenation.	Continuously rocked by a rocker device	NS	10 mL
Drew, J. (2015) [23]	Colon tissue	Explant placed on wire mesh on 6-well plates with minimal coverage of media over the explant. Cultured under continuous rocking motion within incubator.	Continuously rocked by a rocker device	Chamber was flushed with 95%O_2_/5% CO_2_ for 10 min at each culture point. Cultured for 14 h	3.5 mL
Paish, H. (2019) [24]	Liver tissue	BioR plate used for ex-vivo tissue culture. The BioR plate has 2 wells which are interconnected by a common channel in order to facilitate cross communication between the wells. Plate cultured on a rocking bioreactor platform. Tissue cultured on trans-well support	Continuously rocked by a rocker device	Media replenished daily	3 mL

**Table 3:** Characteristics of studies utilising dynamic perfusion culture.

Author	Tissue Cultured	Perfusion Flow Rate	Timing of Flow	Pump Mechanism	Volume of Chamber	Outflow Management
Surowiec, S. (2000) [25]	Saphenous vein	100 mL/min	Intermittent	Peristaltic pump	500 mL	NS
Strehl, R. (2005) [26]	Articular cartilage from femoral trochlear region	1 mL/h	Intermittent	Peristaltic pump	N/A	Passive
Cheah, L. (2010) [27]	Heart tissue	120 mL/min	Intermittent	Peristaltic pump	400 mL	Active
Midwoud, P. (2011) [28]	Liver tissue	10 mL/min	Continuous	Peristaltic pump	25 μL	Passive
Atac, B. (2013) [29]	Juvenile prepuce	7–70 mL min	Intermittent	Micropump	500 mL	Active
Astolfi, M. (2016) [30]	Ovarian and prostate tissue	20 μL/min	Intermittent	Micropipette pump	500 μL	Active
Perrard, M. (2016) [31]	Testicular tissue	NS	NS	NS	8 mL	Passive
Muraro, M. (2017) [32]	Breast tissue	0.3 mL/min	Continuous	Syringe pump	8 mL	Passive
Piola, M. (2017) [33]	Saphenous vein	40–240 mL/min	Intermittent	Peristaltic pump	50 mL	Passive
Bower, R. (2017) [34]	Laryngeal, oropharyngeal or oral cavity tissue	2 μL/min	Intermittent	Syringe pump	NS	Passive
Rodriquez, A. (2019) [35]	Rectal tissue	1.5 mL h^−1^	Continuous	Syringe pump	480 μL	Active

NS—Not specified.

**Table 4:** Description of studies with successful culture of ex vivo human tissue utilising dynamic physiological culture.

	Author	Tissue Cultured	Condition of Tissue	Duration Cultured Ex-Vivo	Tissue Acquisition	Duration of Ischaemic Cellular Injury	Tissue Sample Preparation	Tissue Sample Thickness	Viability Assessments	Preservation of Native Function within Tissue	Drug Delivery Assessed	Description
Gastrointestinal System	Duray, P. (2005) [21]	Tonsillar tissue	Normal	12 days	Tonsillectomy	Processed within 5 h of surgery	Manual dissection (Scalpel)	2 mm cubes	Histological assessment	N/A	N/A	Inoculating of tonsils with Borelia burgoferfi with subsequent confirmation of bacterial proliferation with PCR.
	Midwoud, P. (2011) [28]	Liver tissue	Normal	24 h	Redundant donor tissue after split-liver transplantation	N/A	Microtome (Krumdieck tissue slicer)	100 μm-thickness	Bile acid synthesis by CYP71, Trends in transaminase level	Bile acid synthesis	Drug metabolism	Co-cultured of liver with intestinal slice (multi tissue culture)
	Paish, H. (2019) [24]	Liver tissue	Normal	6 days	Hepatectomy	2 h	Vibratome (Leica VT1200S)	250-μm-thickness	Albumin secretion	Albumin secretion	Drug induced fibrosis (TGFβ1 + PDGFββ) and anti-fibrsosis drug (ALKi)	Profiling immune cell populations during ex vivo culture. Platform for antifibrosis drug screen
	Rodriquez, A. (2019) [35]	Colon tissue	Diseased (rectal cancer)	3 days	Colectomy	N/A	Vibratome (Leica VT1200S)	250 μm-thick	Hoechst staining	N/A	Yes -Assessed by apoptosis and proliferation rates	Comprehensive drug screen (3 regimes tested)
Musculoskeletal System	Strehl, R. (2005) [26]	Articular cartilage from femoral trochlear region	Normal	56 days	Surgical specimens	Processed immediately after surgery	Stainless steel punch (MiltexInstruments)	Full thickness cylindrical explants of 3 mm × 3 mm	Histological assessment of morphology and IHC staining for extracellular matrix. Mitotic index determined.	N/A	N/A	Determining hyaline cartilage composition with sub population changes over time. Proliferation capacity ex vivo
	Aiyangar, A. (2014) [17]	Trabecular bone (L1 vertebrae)	Normal	N/A	Post mortem acquisition	N/A	Diamond coated band saw	5 mm in height and 10 mm in diameter	N/A	Mechanical properties (pressure distribution)	N/A	Radiological assessment (CT) of vertebral body volumetry.
	Walter, B. (2014) [18]	Intervertebral Disc	Normal	21 days	Lumbar Cadaveric samples	N/A	Histologic band saw (Exakt310)	N/A	Viability assays (MTT & DAPI staining)	Mechanical properties (Mechanotransduction)	N/A	Comprehensive assessment of pressure application and subsequent tissue response
	Rosenzweig, D. (2016) [15]	Intervertebral Disc	Normal	10 days	Post mortem acquisition	Less than 4 h	High-speed drill (Foredom)	Varying disc height—0.8–1.65 cm	Viability assays (MTT & DAPI staining)	Mechanical properties (Mechanotransduction)	N/A	Detailed assessment of pressure application with a intermittent cycle with a recovery period
	Margolis, L. (1999) [19]	Prostatic tissue	Normal	28 days	Transurethral prostatectomy/needle biopsy	N/A	Manual dissection (dissection)	1 × 1 mm blocks	Histological assessment	N/A	N/A	Determining PSA expression ex-vivo
	Ladd, M. (2009) [16]	Juvenile prepuce	Normal	6 days	Routine circumcision	N/A	N/A	N/A	Histological and IHC assessments	Mechanical properties of skin (collagen staining)	N/A	Tensile strength assessment of skin
Genito-Urinary System	Atac, B. (2013) [29]	Juvenile prepuce	Normal	14 days	Routine circumcisions	Immediately after surgery	N/A	N/A	Histological assessment. IF staining for apoptosis and proliferation.	N/A	N/A	Multi organ culture-skin and hair
	Perrard, M. (2016) [31]	Testicular tissue	Normal	60 days	Orchidectomy	N/A	N/A	20 to 50 mm3 of isolated seminiferous tubule segments	Histological assessment for morphology	Spermatogenesis	N/A	Evaluating spermatogenesis ex vivo
	Astolfi, M. (2016) [30]	Ovarian and prostate tissue	Diseased (Ovarian and prostate cancer)	8 days	Surgical resection	Processed within 3 h of surgery	Vibratome	300 micrometre slices	Staining with liability dyes–CTG and PI	N/A	Yes, carboplatin used.	Personalised drug screen
Cardiovascular System	Surowiec, S. (2000) [25]	Saphenous vein	Normal	96 h	Segments obtained following coronary artery bypass grafts	Immediately after surgery	Manual dissection (dissection)	Average vessel length of 5 cm was used for these experiments (range 3–10 cm)	Histological assessment of morphology and BrdU staining for proliferation	Dynamic response to stimulus-relaxation and contraction	Yes, arterenol + carbachol	Determining tissue response to external stimuli
	Cheah, L. (2010) [27]	Heart tissue	Normal	5 h	Cardiac surgery	Placed in the perfusion chamber within 60 min of surgery	Manual dissection (dissection)	N/A	Viability assays and LDH release	Contractile function	N/A	Response to electrostimulation within cardiac tissue
	Piola, M. (2017) [33]	Saphenous vein	Normal	7 days	Segments obtained following coronary artery bypass grafts	Immediately after surgery	Manual dissection (Dissection)	N/A	Histological assessment for morphology. IHC for proliferation index	Dynamic response to stimuli	N/A	Determining the effects of haemodynamic stimuli on vessel patency
	Muraro, M. (2017) [32]	Breast tissue	Diseased (Breast cancer)	21 days	Surgical resection	Immediately after surgery	Vibratome (McIlwain Tissue Chopper device)	2 × 2 × 2 mm fragments	Histological assessment of morphology. Proliferation index assessed and immune profiling of cancer.	N/A	Yes, anti-oestrogen therapy	Tumour microenviroment preserved -immune checkpoint blockade therapy trailed
Miscellaneous	Licato, L. (2001) [20]	Melanoma tissue	Diseased	14 days	Surgical resection	Immediately after surgery	Manual dissection (Scalpel)	1–2 mm^2^	Histological assessment of morphology. IHC and immune profiling of tumour	N/A	N/A	Immune profiling of tumour
	Ferrarini, P. (2013) [22]	Bone marrow	Diseased (Multiple myeloma)	24 days	Bone marrow biopsy	N/A	N/A	2–3 mm^3^	Histological assessment of morphology	Analysis of supernatants of tumour secretions	Yes, Bortezomib	Assessment of tumour biology determined by tumour secretions into the media
	Bower, R. (2017) [34]	Laryngeal, oropharyngeal or oral cavity tissue	Diseased (Head and neck squamous cell carcinomas tissue)	48 h	Surgical resection	Within 90 min of excision	Manual dissection (Scalpel)	N/A	Histological assessment of morphology. Viability assay with proliferation and cell death via flow cytometry	N/A	N/A	Determining viability trends ex vivo
	Riley, A. (2019) [36]	Thyroid tissue	Diseased (Thyroid cancer)	24 h	Surgical resection during thyroidectomy	Within 60 min of surgical excision	Vibratome (Leica VT1200S)	5 mm in diameter	Histological assessment of morphology. Viability assay. IHC for proliferation	Hormone production -thyroxine release	N/A	Determining preservation of endocrine function

N/A—Not assessed, CT—Computerised Tomography, IHC—Immunohistochemistry, IF—Immunofluorescence.

## Data Availability

Data from this systematic review have been obtained from previously published articles. Data supporting reported results are available from the corresponding author upon request.

## Supplementary Materials

The following are available online at https://www.mdpi.com/article/10.3390/cancers13122870/s1, Table S1: Description of tissue specific media and supplementation regime.
