# Supplementary material for: Dynamic Physiological Culture of Ex Vivo Human Tissue: A Systematic Review
Source: Cancers (Basel). 2021 Jun 8;13(12):2870. doi: 10.3390/cancers13122870 (PMC8229413; doi:10.3390/cancers13122870)
Supplement: Supplementary file 1 [file cancers-13-02870-s001.zip › cancers-1221306-supplementary.pdf]

Supplementary materials

Supplementary Table S1 – Description of tissue specific media and supplementation regime

Author	Tissue cultured	Normal or diseased	Baseline media used	Antibiotics prophylaxis	Growth factors supplementation	Amino acid supplementation	Steroid supplementation	Other
Duray, P. (2005)	Tonsillar tissue	Normal	RPMI 1640 medium	NS	NS	NS	NS	NS
Midwoud, P. (2011)	Liver tissue	Normal	NS	NS	NS	NS	NS	NS
Drew, J. (2015)	Colon tissue	Normal	RPMI 1640 medium	NS	NS	NS	NS	NS
Paish, H. (2019)	Liver tissue	Normal	Hank's balanced salt solution	NS	TGFβ1 (3ng/ml)+ PDGFββ (50ng/ml)	NS	NS	NS
Rodriquez, A. (2019)	Rectal tissue	Diseased (Metastatic rectal cancer)	DMEM/F12	NS	NS	NS	NS	NS
Strehl, R. (2005)	Articular cartilage from femoral trochlear region	Normal	Serum-free DMEM/F12+	Gentamycin (50mg/ml)+ fungizone (2mg/ml)	NS	NS	NS	HEPES buffer + Ascorbic acid (50µg/mL)
Aiyangar, A. (2014)	Trabecular bone (L1 vertebrae)	Normal	Sterile saline	NS	NS	NS	NS	NS
Walter, B. (2014)	Intervertebral Disc	Normal	High glucose Dulbecco's Modified Eagle Medium	1% penicillin/streptomycin, 0.5% fungizone	10% fetal bovine serum	NS	NS	Ascorbic acid (50µg/mL), primocin (1:500)
Rosenzweig, D. (2016)	Intervertebral Disc	Normal	NS	NS	NS	NS	NS	NS
Margolis, L. (1999)	Prostatic tissue	Normal	RPMI-1640	Fungizone (2.5 pg./ml.), timentin (10 pg.Ipl)	15% fetal calf serum	Glutamine (100 pg./ml)	NS	NS
Ladd, M. (2009)	Juvenile prepuce	Normal	High-glucose Dulbecco's modified Eagle medium	NS	10% fetal bovine serum	NS	NS	PSA 2%
Atac, B. (2013)	Juvenile prepuce	Normal	High-glucose Dulbecco's modified Eagle medium	100 units/ml penicillin + 100ugml streptomycin	10% foetal calf serum	1% Glutamine	NS	NS

Perrard, M. (2016)	Testicular tissue	Normal	Dulbecco's modified Eagle medium	NS	NS	10 µg/ml Insulin	Testosterone (10-7M)	Hepes buffer (15mM), vitamin C (10-4 M), retonoic acid (3.3 x 10-7 M), Sodium bicarbonate, Transferrin (10ug/ml), Vitamin E (10 µg/ml), retinol (3.3 x 10-7M), pyruvate (10-3 M)
Astolfi, M. (2016)	Ovarian and prostate tissue	Diseased (Ovarian and prostate cancer)	Hank's Buffered Saline Solutio	Gentamicin(55 mg L−1), Amphotericin B (600µgL−1)	Fetal Bovine Serum 10%	NS	NS	NS
Muraro, M. (2017)	Breast tissue	Diseased (Breast cancer)	Dulbecco's modified Eagle medium	NS	Autologoushuman serum 10%, Epidermal Growth Factor (25 ng/mL, 1% Kanamycin sulfate	1% Glutamine	NS	1% HEPES 1M , N-AcetyL-Cysteine 1 mM , Nicotinamide (10 mM),
Surowiec, S. (2000)	Saphenous vein	Normal	Low- glcose Dulbecco's modified Eagle medium	10,000 U/ml penicillin G, 10,000mg/ml streptomycin sulfate, 25mg/ml amphotericin	NS	Glutamine (200 mM)	NS	NS
Cheah, L. (2010)	Heart tissue	Normal	Krebs-Henseleit buffer with 5mM glucose	100 Unit mL-1 penicillin and 0.1 mg ml-1 streptomycin	NS	NS	NS	Krebs-Henseleit buffer (KH, 118 mM NaCl, 25 mM NaHCO3,4.8 mM KCl, 1.2 mM KH2PO4, 1.2 mM MgCl2and 2.5 mMCaCl2)
Piola, M. (2017)	Saphenous vein	Normal	Dulbecco's modified Eagle medium	1% Penicillin/Streptomycin	10 % Fetal Bovine Serum	1% L-Glutamine	NS	NS
Licato, L. (2001)	Melanoma tissue	Diseased	Dulbecco's modified Eagle medium	100 I-~g/ml streptomycin, 100 txg/ml kana- mycin, 100 U/ml ampicillin, and 50 ~g/ml gentamicin	10 % Fetal Bovine Serum	2mM Glutamine	NS	20 mM N-2-hydroxyethylpiperazine-N'-2-ethane-sulfonic acid buffer, 2.5 ~g Fungizone
Ferrarini, P. (2013)	Bone marrow	Diseased (Multiple myeloma)	RPMI 1640 medium	NS	10 % Fetal calf Serum	NS	NS	NS
Bower, R. (2017)	Laryngeal, oropharyngeal or oral cavity tissue	Diseased (Head and neck squamous cell carcinomas tissue)	dulbecco's modified Eagle medium	penicillin (0.1 U/ml) /streptomycin (0.1 mg/ml)	fetal Bovine Serum 10%	2mM Glutamine	NS	HEPES Buffer, 4.5 g/l glucose, .1 mM NEAA (nonessential amino acids), 2.5 µg/ml Amphotericin B,

NS – Not specified
